# A spatiotemporal dataset for integrated assessment and modelling of crop-livestock integration with the MAELIA simulation platform

**DOI:** 10.1016/j.dib.2021.107022

**Published:** 2021-04-01

**Authors:** Rui Catarino, Olivier Therond, Jérémy Berthomier, Christian Bockstaller, Michael Curran, Maurice Miara, Emmanuel Mérot, Antoine Messean, Renaud Misslin, Paul Vanhove, Florence Van Stappen, Didier Stilmant, Jean Villerd, Frederique Angevin

**Affiliations:** a Université de Lorraine, INRAE, LAE, 28 Rue de Herrlisheim, F-68000 Colmar, France; b Chambre d'agriculture Pays de la Loire, GEDA de Pouzauges/La Chataigneraie, France; c Department of Socio-Economic Sciences, FiBL, Frick, Switzerland; d INRAE, Eco-Innov, F-78850 Thiverval, Grignon, France; e Centre Wallon de Recherches Agronomiques, CRA-W, 9 Rue de Liroux, 5030 Gembloux, Belgium

**Keywords:** Agroecological transition, Decision support system, Multi-criteria analysis, Participatory design, Agent-based modelling, Social-ecological system

## Abstract

The general purpose of the primary and secondary data available in this article is to support an integrated assessment of scenarios of crop-livestock integration at the territorial level i.e. of exchanges between arable and livestock farms. The data is a result of a research collaboration between the scientist from INRAE, agricultural advisers from Chamber of Agriculture of Pays de la Loire (CRAPL) and a collective of five arable and two livestock farmers located in the district of Pays de Pouzauges (Vendée department, western France). All participants formed part of the DiverIMPACTS project (https://www.diverimpacts.net/) that aims to achieve the full potential of diversification of cropping systems for improved productivity, delivery of ecosystem services and resource-efficient and sustainable value chains in Europe. The first dataset corresponds to the inputs of MAELIA (http://maelia-platform.inra.fr/), a spatial agent-based simulation platform that was used to support an iterative design and assessment of scenarios to redesign cropping systems. The second dataset corresponds to the outputs of MAELIA simulations and the associated indicators at the farm, group and territory level. The data comprise multiple shape and csv files characterizing the edaphic-climatic heterogeneity of the territory and cropping systems, farmers’ crop management rules (IF-THEN rules) and general information about the farms (e.g. crops, agricultural equipment, average crop yields). Data is reported for the baseline situation and three exchange scenarios containing different innovative cropping systems co-designed by scientists, agricultural advisers and the farmers. The data presented here can be found in the Portail Data INRA repository (https://doi.org/10.15454/3ZTCF5) and were used in the research article “Fostering local crop-livestock integration via legume exchanges using an innovative integrated assessment and modelling approach: MAELIA” [1].

## Specifications Table

**Table:** 

Subject	Agricultural Sciences
Specific subject area	Territorial crop-livestock systems
Type of data	Shape (*.shp) and csv files
How data were acquired	- Primary data were collected for the period from July 2014 to September 2018. Farmer surveys (general information about the farms and farmers’ decision management rules) were realised in March and April 2019. - Field boundaries correspond to the French Land Parcel Identification System (LPIS) of the year 2017. - Soil data was collected from the Geographical Database of French Soils (BDGSF) at a scale of 1:1.000.000, and improved with soil analysis from the farmers. - Meteorological data was collected from SAFRAN dataset (8 km × 8 km) of Météo France. - Simulation data (second dataset) corresponds to the outputs of MAELIA.
Data format	Raw and simulated.
Parameters for data collection	Farmer surveys were realised face-to-face based on a uniform template.
Description of data collection	Data were collected for seven farms, 195 fields, 70 crop rotations and 15 crops. Farmers’ decision management rules and general information about the farms (e.g. crops, equipment, average crop yields) was collected via surveys. Each farmer was interviewed individually.
Data source location	Institution: INRAE Region: district of Pays de Pouzauges, Vendée Country: France from 46.7°N to 46.9°N latitude and, 0.7°W to 0.9°W
Data accessibility	Repository name: Data INRAE Title: *Replication Data for TCLS study in Vendee, France* Data identification number: https://doi.org/10.15454/3ZTCF5 Direct URL to data: https://doi.org/10.15454/3ZTCF5
Related research article	Catarino, R., Therond, O., Berthomier, J., Miara, M., Mérot, E., Misslin, R., Vanhove, P., Villerd, J., Angevin, F., 2021a. Fostering local crop-livestock integration via legume exchanges using an innovative integrated assessment and modelling approach based on the MAELIA platform. Agric. Syst. 189, 103066. https://doi.org/10.1016/j.agsy.2021.103066

## Value of the Data

• This dataset offers a unique set of detailed and spatially explicit data on 7 farms including a description of crop management strategies through decision rules.
• This dataset can be used by researchers to perform a multi-criteria assessment of crop diversification scenarios considering socio-ecological and economic dynamics.
• As MAELIA is an open-source platform (http://maelia-platform.inra.fr/) this dataset can be used to define and simulate new scenarios.
• This data allows evaluating self-sufficiency, sustainability and vulnerability of cropping systems from field and farm to group of farms levels.
• This data permits to obtain several socio-economic (e.g. gross margin) and environmental indicators (e.g. nitrogen use and quantity of pesticide active ingredient applied) to evaluate performance at various scale (from field to territory).

## 1 Data Description

We provide two datasets in this paper that were used in the research article “Fostering local crop-livestock integration via legume exchanges using an innovative integrated assessment and modelling approach: MAELIA” [1]. Firstly, a complete dataset of the inputs necessary to run MAELIA (http://maelia-platform.inra.fr/), a high-resolution agent-based platform for IAM (Integrated Assessment Modelling) of agricultural landscapes considered as socio-agroecological systems [2]. Secondly, the raw outputs of MAELIA simulations and the respective indicators at the farm, group and territory level. Overall, it corresponds to an integration of generic data and local knowledge, as well as the data for the simulated baseline situation and the three scenarios considered, as described below in detail.

### 1.1 MAELIA input dataset

This dataset includes spatially explicit data, in the format of shapefiles (*.shp), concerning the administrative divisions, soil mapping units, meteorological zones (8 × 8 km) and, for each farm, field blocks (herein islets) and fields. To avoid any sort of identification, we have anonymised fields that could directly be linked to the farmer. It also includes local and expert-based data that were gathered through direct collaboration with the parties involved in the study, such as the farmers and advisors of local chamber of agriculture. Lastly, it contains the observed crop sequences within each field, crop management strategies described through decision rules, equipment used, production (yield) and economic information (prices and costs). The description of each variable, the unit of measurement, the nature of data and respective units are presented in Tables 1, 2, 3, 4, 5, 6, 7 and 8. Below we explain each of different data files present in the MAELIA input dataset, and the nature of data, that is available at Portail Data INRA repository (https://doi.org/10.15454/3ZTCF5):

• Administrative divisions: Spatial data containing information concerning the second- (ADM2, *department.shp*) and forth (ADM4, *communes.shp*) -order French administrative divisions, referent to provinces and communes respectively. These data serve as a basis for delineating the territory.
• Water catchment area: General information regarding the characteristics of the water catchment area (*ZH.shp*).
• *Soil mapping units* (SMUs) and detailed quantitative soil data (*soils.shp*). Each SMUs of the 1:1 000 000 French soil map [3] were tagged to the dominant Soil Typological Unit (STU). Then pedotransfer rules [4] were used to transform qualitative data of STU into quantitative values describing characteristics and properties of the corresponding soil. Finally, this soil data were improved using soil analyses provided by the farmers.
• Agricultural fields: The French Land Parcel Identification System (LPIS) (v2017), a geographical database [5], [6], was used to provide the boundaries of fields and field blocks (islets) of each farm investigated. For each of the three land-use scenarios, field data is composed by two different geographical units to represent farm's agricultural area: islets (*islets_base.shp, islets_coexistence.shp, islets_complementarity.shp* and *islets_synergetic.shp*) and parcels (*parcels_base.shp, parcels_coexistence.shp, parcels_complementarity.shp* and *parcels_synergetic.shp*). Islets are formed by one or a set of contiguous georeferenced parcels belonging to the same farm and delimited by natural (e.g. hedges, ditches) or artificial elements of the landscape (e.g. road). An islet is considered homogeneous in weather and soil conditions. Parcels represent the georeferenced agricultural area within the islets, where crops are grown and the farmers perform their management activities. Each arable parcel of the farm contains a defined vegetal cover sequence (including crop, cover crop and/or grasslands), used to simulate the vegetal cover dynamics along years. The information is provided whether the islet can be irrigated or not.
• Weather data series: It describes the weather zones (*polygonesMeteoFrance.shp*) based on the grid of points (8 × 8km) provided by SAFRAN [7]. For each year, an individual file containing daily temperature (minimum, medium and maximum), rainfall and evapotranspiration, details the quantitative climate data (*2005.csv* to *2017.csv*).
• Decision rules (*DecisionRules.csv*), formalised through nested IF-THEN-ELSE rules, represent crop management strategies underpinning the triggering of technical operations. A strategy (called hereafter ITK for technical itinerary) corresponds to a set of decision rules for triggering a set of technical operations. It has to be defined for each crop and each situation (soil type, crop sequence…) that determines crop management. In MAELIA, these decision rules, are used to simulate spatiotemporal dynamics of tillage, sowing, fertilization, irrigation, pesticide applications and harvesting. For each technical operation, the decision rules describe which crop (including crop development stage), soil (eg. maximum humidity), or climate conditions (eg. maximum rainfall or temperature) are necessary to trigger each cultural operation and the temporal window during which it can be done. Multiple temporal windows with respective conditions can be defined for each technical operation, which permits to relax constraints along the time. In MAELIA, the daily spatiotemporal distribution of cultural operations over the farm's fields is subject to the time necessary to perform each cultural operation and the spatial distribution and size of fields. For further information see [2], [8], [9].
• Crop parameters: The crop species file (*crop_parameters.csv*) contains the parameters for plant growth (see Table 4 for a detailed description).
• The irrigation equipment (*irri_equi.csv*) contains the equipment used for irrigation (see Table 5 for a detailed description).
• Economic information. The *economic_info.csv* file contains information regarding the crop prices and premiums, milk prices, feeding costs, water costs, and average variable costs for each crop. These data were provided by agricultural advisors from the CRAPL for 2015, 2016 and 2017. For the remaining simulated period (2005 to 2014) they were extrapolated using the agricultural producer price index for each year for each input and output [10].

**Table 1:** Description and respective definition of all parameters used in the shapefile dataset, including administrative divisions, water catchment area, soil mapping units, agricultural fields and weather data series. The table shows the denomination of different variables, together with a respective full description, the type of variable and their units.

Variable	Description	Type	Units
*department.shp*			
code_insee	Department INSEE code	String	-
CODE_DEPT	Department code	String	-
*communes.shp*			
NOM	District name	String	-
code_insee	District INSEE code	String	-
DEPART	Department name		
*soils.shp*			
STU_DOM	Identifier of the dominant *Soil Typological Units* (STUs) of the 1:1 000 000 French soil map	String	-
ID_ZH	Identifier of the water catchment area(s) or water catchment area levels	String	-
ZONE_PEDO	Identifier of a common soil area (e.g. pln_sndy for a sandy plain). It is up to the user to fill this field (if unknown “all” is used).	String	-
ID_SOL	Unique soil type identifier per ZH (composed by ID_ZH x STU_DOM x ZONE_PEDO)	Double	-
CSTRU	Note on soil structure quality estimated by experts Scale: 0 (roots cannot access to the soil) to 1 (roots can access to the whole soil). Standard value 0.9	Double	-
PIRM	Soil infiltrability (permeability) in the surface horizon (P1)	Double	mm/day
ARG_OC	Soil total clay rate	Double	%
PRO_OC	Total soil depth	Double	cm
P1	Horizon depth in horizon explored by roots	Double	cm
P2	Second horizon layer depth	Double	cm
P3	Third horizon layer depth. Optional data	Double	cm
P4	Forth horizon layer depth. Optional data	Double	cm
ARG1	Percentage of clay in P1	Double	%
ARG2	Percentage of clay in P2	Double	%
ARG3	Percentage of clay in P3	Double	%
ARG4	Percentage of clay in P4	Double	%
CX1	Percentage of stone/gravel in P1	Double	%
CX2	Percentage of stone/gravel in P2	Double	%
CX3	Percentage of stone/gravel in P3	Double	%
CX4	Percentage of stone/gravel in P4	Double	%
DAH1	Apparent density in P1	Double	g/cm3
DAH2	Apparent density in P2	Double	g/cm3
DAH3	Apparent density in P3	Double	g/cm3
DAH4	Apparent density in P4	Double	g/cm3
RUPH1	Available water capacity in P1	Double	mm
RUPH2	Available water capacity in P2	Double	mm
RUPH3	Available water capacity in P3	Double	mm
RUPH4	Available water capacity in P4	Double	mm
KSTA1	Soil hydraulic conductivity in P1	Double	mm/day
KSTA2	Soil hydraulic conductivity in P2	Double	mm/day
KSTA3	Soil hydraulic conductivity in P3	Double	mm/day
KSTA4	Soil hydraulic conductivity in P4	Double	mm/day
*ZH.shp*			
ID_ZH	Identifier of the water catchment area(s) or water catchment area levels	String	-
EU_CD	European identifier of watershed (if unknown state 0)	Double	-
EU_CD_exut	European identifier of watershed outflow (if unknown state 0)	Double	-
PERCENTAGE	Percentage of the water catchment area present in the territory	Double	-
ID_ND_EXUT	National identifier of watershed outflow (if unknown state 0)	Double	-
*islets_base.shp, islets_coexistence.shp, islets_complementarity.shp and islets_synergetic.shp*			
ID_EXPL	Identifier of the farm	String	-
ID_ILOT	Islet identifier	String	-
ID_SOL	Identifier of the associated soil type	String	-
ID_ZH	Identifier of the water catchment area(s) or water catchment area levels	String	-
MATERIEL	Irrigation equipment identifier (if CARACT_IRR = O)	Double	-
PENTE_MOY	Average islet slope	Integer	%
*parcels_base.shp, parcels_coexistence.shp, parcels_complementarity.shp and parcels_synergetic.shp*			
SEQUENCE	Crop sequence (or rotation)	String	-
CULT_REF	Initial cultivation of the parcel	String	-
ID_EXPL	Identifier of the farm	String	-
ID_ILOT	Identifier of the islet	String	-
ID_PARCELL	Identifier of the field, also indicates the islet to which it belongs	String	-
ID_SDC	Identifier of the crop sequence type	String	-
CARACT_IRR	Indicates if the field is irrigable (O, yes; or N, no)	String	-
POURCENTAG	Ratio of field area in the islet area	String	-
SURFACE	Area of the field	Double	Ha
INDEX_DEP	Starting index in the sequence	Integer	-
*polygonesMeteoFrance.shp*			
POSY	Y distance to the centre of the polygon	Integer	-
POSX	X distance to the centre of the polygon	Integer	-
ID_PDG	Weather polygon identifier	String	-
ALTI_MOY	Average altitude of the weather zone	Integer	m

**Table 2:** Description of columns heading belonging to DecisionRules.csv file. The table enumerates and labels the different columns and provides a respective description. The actual decision rules necessary to trigger each crop management operations are presented in Table 3a-3d.

Column nb	Label	Description
1	NOM_ITK_AFFICHAGE	Identifier of decision rule parameters. These names are used in MAELIA's outputs.
2	*	Associated unit
2-n	Identifiers of ITKs	Value of decision rule parameters for an ITK

**Table 3:** Description of the characteristics of each cropping system for column NOM_ITK_AFFICHAGE (rows 1-9) present in DecisionRules.csv file. A respective explanation of each label, including units, is provided.

Row nb	Designation	Operation technique	Description	Units
1	NOM_ITK_AFFICHAGE		Identifier of decision rule parameters. These names are used in MAELIA's outputs.	-
2	ID_ITK		Identifier of a crop management strategy (ITK, technical itinerary) i.e. a set of decision rules parameters for a crop in a management situation.	-
3	IDS_SDCS		List of crop sequences in which the respective ITK can be applied, to be separated by “|'”.	-
4	IDS_SDCS_CLASS		Identifier of the group (or class) of the different IDS_SDCS, to be separated by “|”.	-
5	ID_ESPECE		Identifier of the crop’ species concerned by the respective ITK	See *crop_parameters.csv*
6	MATERIEL		Identifier of the irrigation equipment or 'NA' if no irrigation equipment is used	See *irri_equi.csv*
7	ZONE_PEDO		Identifier of a common soil area (e.g. pln_sndy for a sandy plain) in which the respective ITK can be applied, to be separated by “|”.	See *soils.shp*
8	ZONE_PEDO_CLASS		Identifier of the group (or class) of the different ZONE_PEDO, to be separated by `|'.	-
9	IS_CULTURE_HIVER		Boolean distinguishing winter crops from others. This information is necessary for the temporal chaining of crops in a field	O (yes) or N (no)

**Table 4:** Description of the common nomenclature, and units, of rules for carrying out the technical operations (TO) for column NOM_ITK_AFFICHAGE (rows 10-106) present in DecisionRules.csv file. The following TOs are present: PREPA (P), REPRISE (R), SEMIS (S), BINAGE (B), RECOLTE (H), IRRIGATION (I), FERTI (F) and PHYTO (PY). If NB_SOUS_PERIODES>1, values in the following fields are separated by '|', and a value is defined per sub-period.

Row nb	Designation	Operation technique	Description	Units
10, 21, 32, 44, 53, 65, 89, 99	IS_TO	P, R, S, B, H, I, F, PY	Activates the technical operation TO	O: if TO is present N: if TO is NOT present
11, 22, 34, 46, 56, 68, 90, 102	*TO_*NB_SOUS_PERIODES	P, R, S, B, H, I, F, PY	Number of sub-periods to be considered	[1; + ∞[
12, 23, 33, 45, 54, 91, 100	*TO_*TEMPS	P, R, S, B, H, F, PY	Working time expressed in number of hectares completed in 1 hour for the technical operation TO.	ha/h
13, 24, 35, 47, 57, 69, 92, 103	*TO_*DEBUT	P, R, S, B, H, I, F, PY	List of sub-period start dates. To be separated by “|”	Day [1-366]
14, 25, 36, 48, 58, 70, 93, 104	*TO_*FIN	P, R, S, B, H, I, F, PY	List of sub-period end dates. To be separated by “|”	Day [1-366]
15, 26	*TO_*JOURS_P-ETP_MOY	P, R	Number of contiguous days to be considered for the condition regarding a threshold of [cumulative precipitation – evapotranspiration]. To be separated by “|”	Day [1-366]
16, 27	*TO_*P-ETP_MIN	P, R	[Rainfall accumulation – Evapotranspiration] above which the OT is postponed. To be separated by “|”	[mm]
17, 28, 39, 60	*TO_*JOURS_PLUIE	P, R, S, H	Number of contiguous days to be considered for the condition regarding a threshold of [cumulative precipitation]. To be separated by “|”	Day [1-366]
18, 29, 40, 61	*TO_*HAUTEURS_PLUIE_MAX	P, R, S, H	Rain accumulation above which the TO is postponed. To be separated by “|”	[mm]
19, 30, 41, 50, 62, 81	*TO_*HUMIDITE_SOL_MAX	P, R, S, B, H, I	Maximum soil humidity above which the TO is postponed. To be separated by “|”	% of the water capacity
20, 31, 42, 51, 63	*TO_*EFFET_RUs	P, R, S, B, H	Depth of soil tillage. NA allows you to specify the absence of soil tillage. To be separated by “|”	NA: no effect on soil texture W1, W2 and W3: 6, 12 and 30 cm respectively
43, 64, 86	*TO_*OPERATEUR	S, H, I	If TO is performed, or not, by the farmer	O: is performed by the farmer N: is outsourced
95, 100	*TO_*JOURS_PLUIE_OBS	F, PY	Number of contiguous days to be considered for the condition regarding a threshold of observed [cumulative precipitation]. To be separated by “|”	Day [1-366]
96, 106	*TO_*HAUTEURS_PLUIE_OBS_MIN	F, PY	Minimum rain accumulation on the n last day and above which the TO is postponed. To be separated by “|”	[mm]

**Table 5:** Description of the specific nomenclature, and units, for carrying out sowing (SEMIS), hoeing (BINAGE), harvest (RECOLTE), fertilisation (FERTI) and application of pesticides (PHYTO). These data is referent to column NOM_ITK_AFFICHAGE (rows 37-108) present in DecisionRules.csv file.

Row nb	Designation	Operation technique	Description	Units
37	SEMIS_JOURS_TMIN	S	Number of contiguous days to be considered for the condition regarding a threshold of [minimum temperature]. To be separated by “|”	[1-366]
38	SEMIS_TMIN_MIN	S	Minimum temperature on the last OT_JOURS_TMIN days that below the SEMIS is cancelled. To be separated by “|”	[°C]
49	BINAGE_EchV_MIN	B	Vegetation threshold from which hoeing can be carried out. To be separated by “|”	Vegetation scale [0,3]
55	RECOLTE_TEMPS_INTERNE	H	Time dedicated to harvesting by the farmer. If 0, the harvest is carried out by an external service provider.	[0,+ ∞[
59	RECOLTE_ECHV_MIN	H	Vegetation threshold from which harvesting can be carried out. To be separated by “|”	Vegetation scale [0,3]
94	FERTI_DOSE/Ha	F	Fertilization quantities provided per period	Kg/ha
97	FERTI_ECHV_DEBUT	F	Physiological stage values at the beginning of fertilization per period/sub-period(s)	Vegetation scale [0,3]
98	FERTI_ECHV_FIN	F	Physiological stage values at the end of fertilization per period/sub-period(s)	Vegetation scale [0,3]
101	PHYTO_DOSE/Ha	PY	Phytosanitary treatment quantities provided per period/sub-period(s)	Dose/ha
107	PHYTO_JOURS_PLUIE_ PREVUES	PY	Number of contiguous days to be considered in the cumulative forecast precipitation condition authorized for the implementation of phytosanitary treatment.	Day
108	PHYTO_HAUTEURS_PLUIE_ PREVUES_MIN	pY	The cumulative amount of expected precipitation above which treatment is suspended.	mm

**Table 6:** Description of the specific nomenclature, and units, for carrying out irrigation (IRRIGATION) in column NOM_ITK_AFFICHAGE (rows 66-88) present in DecisionRules.csv file.

Row nb	Designation	Operation technique	Description	Units
66	IRRIGATION_TD	I	Minimum number of days between irrigation in the field. To be separated by “|”	Days
67	IRRIGATION_DOSE	I	Irrigation quantity per application. To be separated by “|”	mm
68	IRRIGATION_NB_SOUS_PERIODES	I	Number of sub-periods to be considered for irrigation.	[1,+ ∞[
71	IRRIGATION_ECHV_DEBUT	I	Physiological stage value(s) at the beginning of the irrigation period or subperiods. To be separated by “|”	Vegetation scale [0,3]
72	IRRIGATION_ECHV_FIN	I	Physiological stage values at the end of the irrigation period or subperiods. To be separated by “|”	Vegetation scale [0,3]
73	IRRIGATION_JOURS_PLUIE_CUMUL	I	Number of contiguous days to be considered in the cumulative rainfall condition allowed for irrigation purpose. To be separated by “|”	Days
74	IRRIGATION_HAUTEUR_PLUIE_CUMUL_ ANNULATION	I	Rain accumulation allowed on the last day and beyond which irrigation is postponed. To be separated by “|”	mm
75	IRRIGATION_JOURS_PLUIE_SIGNIF	I	Number of contiguous days to be considered in the condition on significant rainfall. To be separated by “|”	Days
76	IRRIGATION_HAUTEUR_PLUIE_SIGNIF_REPORT	I	The rain quantity above which rain is considered significant to postpone irrigation. To be separated by “|”	mm
77	IRRIGATION_JOURS_PLUIE_PREVUES	I	Number of contiguous days to be considered in the cumulative forecast precipitation condition authorized for irrigation purpose. To be separated by “|”	Days
78	IRRIGATION_HAUTEURS_PLUIE_PREVUES	I	The cumulative amount of forecasted precipitation above which irrigation is postponed. To be separated by “|”	mm
79	IRRIGATION_JOURS_P-ETP	I	Number of contiguous days to be considered for the condition regarding a threshold of [cumulative precipitation – evapotranspiration]. To be separated by “|”	[1,366]
80	IRRIGATION_P-ETP_MAX		Maximum rainfall accumulation – ETP (evapotranspiration) allowed on the last days of OT_JOURS__P-ETP_MOY and above which Irrigation is postponed. To be separated by “|”	[mm]
82	IRRIGATION_IS_THEORIQUE	I	Allows irrigation based on a water satisfaction threshold	O (yes) or N (no)
83	IRRIGATION_SIRR1	I	Crop water satisfaction threshold below which irrigation takes place for crop stage 1 (vegetation scale]0,0.4], ie. emergence stage).	Threshold used to manage automatically irrigation according to a threshold of hydric stress [0,1].
84	IRRIGATION_SIRR2	I	Crop water satisfaction threshold below which irrigation takes place for crop stage 2 (vegetation scale]0.4,0.8[, ie. growing stage).	Threshold used to manage automatically irrigation according to a threshold of hydric stress [0,1].
85	IRRIGATION_SIRR3	I	Crop water satisfaction threshold below which irrigation takes place for crop stage 3 (vegetation scale]1,1.2[, ie. flowering stage).	Threshold used to manage automatically irrigation according to a threshold of hydric stress [0,1].
87	IRRIGATION_REPORT_MAX	I	Maximum number of days for postponing irrigation due to rainfall	Days
88	IRRIGATION_GROUPE	I	Irrigation group family. If the irrigator has more surface area to irrigate than can be irrigated during the water turn, an irrigation group system is set up. An irrigation group consists of irrigable plots belonging to the same farmer, on the same administrative drought restriction zone and managed by the same equipment.	-

**Table 7:** Description of rows belonging to ID_ESPECE column in file crop_parameters.csv. This table enumerates and labels the different rows of the crop_parameters.csv file, and provides a respective description including units.

Row nb	Label	Description	Units
1	ID_ESPECE	Identifier of different crops’ species	-
2	RENDEMENT_MOYEN	Average yield	t/ha
3	RENDEMENT_MIN	Minimum yield	t/ha
4	RENDEMENT_OPTIMAL	Potential (or optimal) yield	t/ha
5-7	COULEUR_(R,V,B)	Colour code for display	-
8	Tbase	Base temperature considered in the calculation of the vegetation scale	°C
9	Tmax	Maximum temperature considered in the calculation of the vegetation scale	°C
10	DEGRES_J_LevTbase	Sum of degrees days at emergence, vegetation scale [1.55, …[	°C
11	DEGRES_J_Flor	Sum of degrees days at flowering, vegetation scale = 1	°C
12	DEGRES_J_matPhyTbase	Sum of degree days at physiological maturity, vegetation scale [1.55, …[	°C
13	FREIN	Fraction of the sum of temperature considered during the winter period. Range between 0 and 1. 1 represents the absence of a brake.	[0-1]
14	CRACINE	Sum of necessary degrees days to make the roots reach 1 mm	°C/mm
15	CVIG	Coefficient for the plant growth's potential	[0-1]
16	KMAX	Maximum Kc of the culture	-
17	CSTO	Effect of stoma closure on water stress	-
18	coeff_Fonction_Prod	Shape coefficient of the production function. Variable called in AqYield. It modulates the effect of water stress on yield	-
19-28	ALPHA*	Element for calculating the Kc curve	-
29-44	KC*	Element for calculating the Kc curve	-
45	ZonesClimatiques	List of climatic zones that can support this crop. To be separated by “|”	-

**Table 8:** Description of the table irri_equi.csv that contains the equipment used for irrigation. This table enumerates and labels the different columns of the file, and provides a respective description including units.

Column nb	Label	Field description	Unit
1		Irrigation equipment identifier. Designation must coincide with “MATERIEL” column in islets*.shp.	-
2	Surface irrigable/jour (ha/jour)	Irrigable surface per day	ha/day
3	Travail (h/jour)	Working time per day	hour/day

### 1.2 MAELIA output raw data

The file *MAELIA_crop_raw.csv* provides for each field all variables representing technical and socio-economic aspects for crop production. And the *MAELIA_livestock_raw.csv* provides all variables representing production for livestock farms. Both files were directly provided by the MAELIA platform. The data is shown for the baseline situation and the three scenarios (Coexistence, Complementarity and Synergetic) over 12 years (2005-2017).

The file *Indicators.csv* provides information regarding all indicators representing performance (Energy yield, Protein yield, Gross margin, Economic efficiency, Nitrogen use, Quantity of active ingredients applied and Workload). These data are shown for each farm, group of farms (arable and livestock) and territory, for the baseline situation and the three scenarios (Coexistence, Complementarity and Synergetic) over 12 years (2005-2017).

## 2 Experimental Design, Materials and Methods

The data presented here is linked to a case study belonging to the DiverIMPACTS project (https://www.diverimpacts.net/case-studies/case-study-11-fr.html) that is based on existing and newly developed initiatives related to crop diversification in Europe. The case study is located in the district of Pays de Pouzauges in the Vendée department (western France) and it is formed by seven farms. The seven farms included five arable farms (AFs: AF1, AF2, AF3, AF4 and AF5) and two livestock farms (LFs: LF1 and LF2, with 65 and 110 dairy cows, respectively).

The design-assessment method using MAELIA is supported by an explicit fine-scale representation of the agricultural landscape with multiple biophysical characteristics (e.g. soil and weather), populated by the seven farmers with individual behaviour and objectives. Two classes of data are necessary to implement the case study in MAELIA: generic data and local expert-based data collected with the relevant stakeholders (see section above “MAELIA input dataset”). In addition, with the farmers and agricultural advisers, we have finely adjusted the parcel boundaries and the respective crop sequence, including the classification of rainfed and irrigated parcels. Soil data were as well amended through soil analysis provided by farmers. With farm surveys realised from March to the end of April 2019, we have collected information about average crop yields, farming practices (pesticide and fertilizer use, tillage and mechanical weed control) and general information about the farm (number of crops, agricultural equipment, etc). For each farmer, the crop management decision rules were collected in parallel via a dedicated farm survey (Supplementary methods in [1] show the template and an example of the survey used to collect decision rules).

Simulations were done using the farm-agent model, incorporated within MAELIA. This model simulates the daily dynamics of technical operations in each field considering their respective soil, climate and plant states and farm-level constraints. The crop management strategy is represented using a set of nested IF-THEN-ELSE statements translating the decision rules obtained from a survey of farmers. The crop yield is modelled with a generic cropping system model (AqYield [11] that simulates in each field the daily interactions between the soil-water cycle, climate, farming practices and crop growth.

Finally, based on the requirements of farmers and advisers we have selected nine criteria and associated indicators to evaluate this case study (see section 2.6 in [1] for a detailed description).

## Ethics Statement

The participants were informed about the purpose of the study and data collection process before the interview started. To all participants was given the power of freedom of choice to decide whether to answer or decline the questions, as well as the possibility of refusing to participate or withdraw from the study while it was in progress. Personal information was handled properly under Directive 95/46/EC on the protection of individuals concerning the processing of personal and on the free movement of such data. Confidentiality of the responses was given assuring that the collected data would be used solely for research purposes. The anonymity of the spatial data of this present dataset is guaranteed via the attribution of a random number to each farmer, farm and field, so no link between these three elements is possible.

## Funding

This research is part of the DiverIMPACTS project funded by the European Union's Horizon 2020 research and innovation programme under grant agreement N. 727482.

## CRediT Author Statement

**Rui Catarino:** Writing original draft, Methodology, Software, Validation, Formal analysis; **Olivier Therond:** Writing review & editing, Conceptualization, Methodology, Validation, Supervision; **Jérémy Berthomier:** Conceptualization, Validation; **Christian Bockstaller:** Validation; **Michael Curran:** Validation; **Emmanuel Mérot:** Conceptualization, Validation; **Antoine Messean:** Project administration; **Renaud Misslin:** Writing review & editing, Software; **Didier Stilmant:** Project administration; **Jean Villerd:** Writing review & editing, Software; **Frederique Angevin:** Conceptualization, Methodology, Validation, Supervision.

## Declaration of Competing Interest

The authors declare that they have no known competing financial interests or personal relationships which have or could be perceived to have influenced the work reported in this article.
